# 超高效液相色谱法同时测定电子烟油中的5种吲哚/吲唑酰胺类合成大麻素

**DOI:** 10.3724/SP.J.1123.2022.10014

**Published:** 2023-07-08

**Authors:** Zhe YANG, Jianxia LYU, Yidi WU, Liwei JIANG, Dongmei LI

**Affiliations:** 国家毒品实验室北京分中心,北京100164; National Anti-Drug Laboratory Beijing Regional Center, Beijing 100164, China

**Keywords:** 超高效液相色谱, 合成大麻素, 吲哚/吲唑酰胺, 电子烟油, 定量分析, ultra performance liquid chromatography (UPLC), synthetic cannabinoids (SCs), indole/indazole amides, electronic cigarette oil, quantitative analysis

## Abstract

合成大麻素是目前世界上滥用最多的新精神活性物质之一,其结构多变,更新迅速,目前已发展至新型第八代吲哚/吲唑酰胺类。近年来与吲哚/吲唑酰胺类合成大麻素相关的案件逐渐增多,在实际案件中对缴获物中合成大麻素的定量分析需求随之增多,但相应的检验鉴定技术仍处于发展阶段。本研究针对电子烟油中5种常见的吲哚/吲唑酰胺类合成大麻素,建立了超高效液相色谱法对其同时进行定量分析测定。实验对流动相的种类、洗脱梯度、柱温、检测波长等色谱条件进行了优化,再结合外标法定量,实现了对5种合成大麻素的定量分析。样品用甲醇提取,在Waters ACQUITY UPLC CSH C18(100 mm×2.1 mm, 1.7 μm)色谱柱上进行分离,柱温35 ℃,流速0.3 mL/min,进样量1 μL,乙腈和超纯水作为流动相进行梯度洗脱,检测波长为290 nm和302 nm。结果表明,采用该方法,5种合成大麻素可在10 min内完全分离,在1~100 mg/L范围内线性关系良好,相关系数(*r*^2^)均可以达到0.9999,检出限为0.2 mg/L,定量限为0.6 mg/L,满足实际样品分析需求。采用1、10、100 mg/L 3个水平的5种合成大麻素混合标准溶液进行精密度试验,日内精密度(*n*=6)均小于1.5%,日间精密度(*n*=6)均小于2.2%。以空白电子烟油为基质样品,在2、10、50 mg/L 3个加标水平下进行加标回收试验,各待测物的平均加标回收率为95.5%~101.9%,相对标准偏差(RSD, *n*=6)为0.2%~1.5%,准确度为-4.5%~1.9%。本方法具有准确、快速、灵敏、分离效果好等优点,适用于电子烟油中5种吲哚/吲唑酰胺类合成大麻素的定量测定,可满足相关鉴定工作的要求,也可为具有相似结构的合成大麻素的液相色谱定量分析提供参考。

## Abstract (en)

Synthetic cannabinoids (SCs), which are considered some of the most widely abused new psychoactive substances available today, are much more potent than natural cannabis and display greater efficacy. New SCs can be developed by adding substituents such as halogen, alkyl, or alkoxy groups to one of the aromatic ring systems, or by changing the length of the alkyl chain. Following the emergence of the so-called first-generation SCs, further developments have led to eighth-generation indole/indazole amide-based SCs. Given that all SCs were listed as controlled substances on July 1, 2021, the technologies used to detect these substances must be quickly improved. Due to the sheer number of SCs, the chemical diversity and the fast update speed, it is challenging to determine and identify the new SCs. In recent years, several types of indole/indazole amide-based SCs have been seized, but systematic research on these compounds remains limited. Therefore, developing rapid, sensitive, and accurate quantitative methods to determine new SCs are of great importance. Compared with high performance liquid chromatography (HPLC), ultra performance liquid chromatography (UPLC) shows higher resolution, better separation efficiency, and faster analysis speeds; thus, it can meet the demand for the quantitative analysis of indole/indazole amide-based SCs in seized materials.

In this study, a UPLC method was developed for the simultaneous determination of five indole/indazole amide-based SCs, including *N*-(1-amino-3,3-dimethyl-1-oxobutan-2-yl)-1-butyl-1*H*-indazole-3-carboxamide (ADB-BUTINACA), methyl 2-(1-(4-fluorobutyl)-1*H*-indole-3-carboxamido)-3,3-dimethylbutanoate (4F-MDMB-BUTICA), *N*-(1-methoxy-3,3-dimethyl-1-oxobutan-2-yl)-1-(5-fluoropentyl)-1*H*-indole-3-carboxamide (5F-MDMB-PICA), methyl 3,3-dimethyl-2-(1-(pent-4-en-1-yl)-1*H*-indazole-3-carboxamido)butanoate (MDMB-4en-PINACA), and *N*-(adamantan-1-yl)-1-(4-fluorobutyl)-1*H*-indazole-3-carboxamide (4F-ABUTINACA) in electronic cigarette oil; these SCs have been detected with increasing frequency in seized materials in recent years. The main factors influencing the separation and detection performance of the proposed method, including the mobile phase, elution gradient, column temperature, and detection wavelength, were optimized. The proposed method successfully quantified the five SCs in electronic cigarette oil via the external standard method. The samples were extracted using methanol, and the target analytes were separated on a Waters ACQUITY UPLC CSH C18 column (100 mm×2.1 mm, 1.7 μm) at column temperature of 35 ℃ and flow rate of 0.3 mL/min. The injection volume was 1 μL. The mobile phase consisted of acetonitrile and ultrapure water, and gradient elution was employed. The detection wavelengths were 290 and 302 nm. The five SCs were completely separated within 10 min under optimized conditions and showed good linear relationships between 1-100 mg/L, with correlation coefficients (*r*^2^) of up to 0.9999. The limits of detection (LOD) and quantification (LOQ) were 0.2 and 0.6 mg/L, respectively. Precision was determined using standard solutions of the five SCs at mass concentrations of 1, 10, and 100 mg/L. The intra-day precision (*n*=6) was <1.5%, and the inter-day precision (*n*=6) was <2.2%. Accuracy was determined by spiking electronic cigarette oil with low (2 mg/L), moderate (10 mg/L), and high (50 mg/L) levels of the five SCs, with six replicates per determination. The recoveries of the five SCs were 95.5%-101.9%, and their relative standard deviations (RSDs, *n*=6) were 0.2%-1.5%, with accuracies ranging from -4.5% to 1.9%. The proposed method showed good performance when applied to the analysis of real samples. It is accurate, rapid, sensitive, and effective for the determination of five indole/indazole amide-based SCs in electronic cigarette oil. Thus, it satisfies the requirements for practical determination and provides a reference for the determination of SCs with similar structures by UPLC.

合成大麻素是模拟大麻植物主要活性成分四氢大麻酚(Δ^9^-THC)的化学作用而合成的一类化合物,通常具有更大的危害性和成瘾性,能产生更为强烈的兴奋、致幻等效果^[1⇓⇓-4]^。自从第一代萘甲酰吲哚类合成大麻素出现后,合成大麻素逐渐成为目前世界上滥用最多的新精神活性物质之一^[5⇓⇓-8]^。合成大麻素的结构一般由4种关键基团组成,即“核心和取代基”、“连接基团”、“环和取代基”及“尾部”,这为结构修饰提供了多处变异位点,例如将取代基如卤素、烷基、烷氧基等添加到芳香环结构中或改变烷基链的长度和排列等^[3,9]^。通过对其分子进行修饰,合成大麻素更新迅速,种类不断增加,自2006年出现第一代萘甲酰吲哚类起,目前已发展至第八代吲哚/吲唑酰胺类合成大麻素^[10,11]^。

2021年5月,公安部、国家卫生健康委员会和国家药品监督管理局联合发布《关于将合成大麻素类物质和氟胺酮等18种物质列入<非药用类麻醉药品和精神药品管制品种增补目录>的公告》,决定正式整类列管合成大麻素类新精神活性物质。公告自2021年7月1日起施行,我国成为全球首个整类列管合成大麻素类物质的国家。因此在实际执法实践中,对可疑毒品缴获物中合成大麻素的检验鉴定需求增加,与之相关的检测技术也亟待提高。此外,国家禁毒委员会先后发布了多份合成大麻素类物质与海洛因的依赖性折算标准,例如1 g 3,3-二甲基-2-[1-(4-戊烯-1-基)-1*H*-吲唑-3-甲酰氨基]丁酸甲酯(MDMB-4en-PINACA)相当于0.2 g海洛因,1 g *N*-(1-氨甲酰基-2,2-二甲基丙基)-1-丁基吲唑-3-甲酰胺(ADB-BUTINACA)相当于0.5 g海洛因,1 g 3,3-二甲基-2-[1-(4-氟丁基)吲哚-3-甲酰氨基]丁酸甲酯(4F-MDMB-BUTICA)相当于5.0 g海洛因。标准发布后,在实际案件中对合成大麻素的定量需求随之增多,因此研究快速、灵敏、准确的新型合成大麻素定量分析方法对执法实践具有重要意义。

吲哚/吲唑酰胺类合成大麻素属于新型的第八代合成大麻素,是一类具有吲哚/吲唑基的酰胺类有机化合物,其种类较新,与之相关的案件数量逐渐增多,但系统性研究较少^[11]^。目前对于可疑毒品缴获物中吲哚/吲唑酰胺类合成大麻素的定量分析研究主要采用气相色谱-质谱法^[12⇓⇓⇓⇓⇓⇓-19]^、液相色谱-质谱法^[20⇓-22]^、核磁法^[18,23,24]^。可疑毒品缴获物中合成大麻素的含量一般为万分之几至百分之几十,在一般实验室中液相色谱法即可满足定量分析测定需求,具有成本低、准确度高、快速高效的优点。因此针对吲哚/吲唑酰胺类合成大麻素开发新的液相色谱定量分析方法是十分有必要的,而与高效液相色谱相比,超高效液相色谱具有更好的分离效能、更高的检测灵敏度以及更快的分析速度。基于此,本文建立了一种采用超高效液相色谱对吲哚/吲唑酰胺类合成大麻素进行定量分析的方法。

MDMB-4en-PINACA、ADB-BUTINACA、4F-MDMB-BUTICA、*N*-(1-金刚烷基)-1-(4-氟丁基)吲唑-3-甲酰胺(4F-ABUTINACA)、2-[1-(5-氟戊基)-1*H*-吲哚-3-甲酰氨基]-3,3-二甲基丁酸甲酯(5F-MDMB-PICA)(结构式见图1)是近年来国内实际案件缴获物中检出次数较多的5种吲哚/吲唑酰胺类合成大麻素,建立相应的定量分析方法,能够更好地服务于实际执法工作。

**图1:**
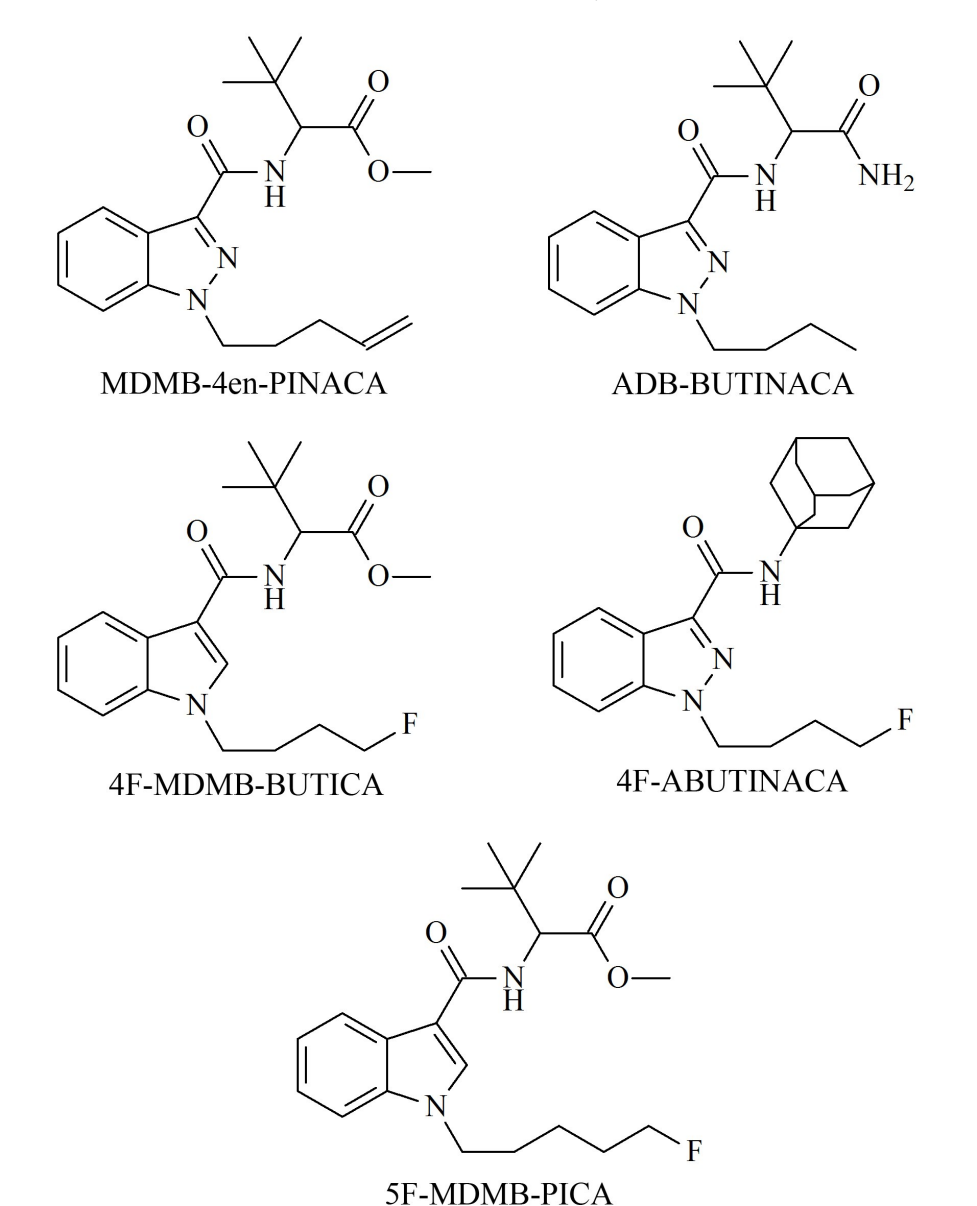
5种合成大麻素分子的结构式

针对上述5种合成大麻素,对其进行色谱分析方法的优化,建立可以同时检测5种合成大麻素的超高效液相色谱方法,并进行定量分析,以期为相关案件缴获物中5种合成大麻素的定量分析工作提供一种新方法,并为具有相似结构的合成大麻素定量检测提供参考。

## 1 实验部分

### 1.1 仪器、试剂与材料

Waters UPLC H-CLASS plus超高效液相色谱仪(沃特世科技(上海)有限公司),配有四元溶剂管理器、样品管理器-针流通模式、光电二极管阵列(PDA)eλ检测器(检测波长为190~800 nm)、Empower 3色谱工作站。

MDMB-4en-PINACA(C_20_H_27_N_3_O_3_,CAS号2504100-70-1, 纯度≥99.5%)、ADB-BUTINACA(C_18_H_26_N_4_O_2_, CAS号2682867-55-4,纯度≥99.5%)、4F-MDMB-BUTICA(C_20_H_27_FN_2_O_3_, CAS号2682867-53-2,纯度≥99%)、4F-ABUTINACA(C_22_H_28_FN_3_O, CAS号1445580-39-1,纯度≥99.5%)、5F-MDMB-PICA(C_21_H_29_FN_2_O_3_, CAS号1971007-88-1,纯度≥99%)均为上海市刑事科学技术研究院与上海原思标物科技有限公司联合研制的标准物质;甲醇、乙腈、磷酸、三乙胺均为色谱纯,购自上海安谱实验科技股份有限公司;实验用水为超纯水,由Milli-Q Reference超纯水机制得。

### 1.2 标准储备液与混合标准溶液的配制

称取各标准物质粉末,分别用甲醇溶解后定容于10 mL容量瓶中,得到5种分析物质量浓度均为1 g/L的标准储备液。量取适量的各分析物标准储备液,配制成5种分析物质量浓度均为200 mg/L的混合标准溶液。取适量混合标准溶液,用甲醇稀释成质量浓度分别为100、50.0、25.0、10.0、5.0、2.5、1.0 mg/L的系列混合标准溶液,备用。

### 1.3 样品溶液的制备

称取电子烟油样本50 mg,加入10 mL甲醇,振荡混匀后超声提取30 min,经0.22 μm有机相滤膜过滤,滤液备用。

### 1.4 色谱条件

Waters ACQUITY UPLC CSH C18色谱柱(100 mm×2.1 mm, 1.7 μm);流动相A为超纯水,B为乙腈,梯度洗脱程序见表1;柱温35 ℃;流速0.3 mL/min;进样量1 μL;4F-MDMB-BUTICA和5F-MDMB-PICA的检测波长为290 nm,ADB-BUTINACA、MDMB-4en-PINACA和4F-ABUTINACA的检测波长为302 nm,采用外标法定量。

**表1:** 梯度洗脱程序

Time/min	Flow rate/(mL/min)	*φ*(A)/%	*φ*(B)/%
0	0.3	50	50
1	0.3	50	50
8	0.3	0	100
10^*^	0.3	50	50

* Rebalance of column; A: ultrapure water; B: acetonitrile.

## 2 结果与讨论

### 2.1 色谱条件的优化

#### 2.1.1 检测波长的选择

通过PDA eλ检测器在190~400 nm内对5种合成大麻素进行扫描,发现ADB-BUTINACA和MDMB-4en-PINACA在208 nm和302 nm处均有较强的吸收,4F-ABUTINACA在209 nm和302 nm处有较强的吸收,4F-MDMB-BUTICA和5F-MDMB-PICA均在218 nm和290 nm处有较强的吸收。由于样品干扰物一般在低波长处有强吸收,且所使用的溶剂甲醇和流动相乙腈-超纯水在低波长处也有较强的吸收,而在波长≥220 nm时吸收较弱^[25⇓-27]^。为了排除有紫外吸收的杂质、溶剂等在低波长处的干扰,且保证5种合成大麻素在分析过程中均有较高的检测灵敏度,最终选择双波长进行检测,即302 nm为ADB-BUTINACA、MDMB-4en-PINACA和4F-ABUTINACA的检测波长,290 nm为4F-MDMB-BUTICA和5F-MDMB-PICA的检测波长。

#### 2.1.2 流动相的选择

分别以乙腈和甲醇为有机相,以65%有机相进行等度洗脱,在302 nm波长下检测时,5种分析物的分离效果如图2所示。当有机相发生变化时,分析物ADB-BUTINACA和4F-MDMB-BUTICA的峰位发生变化,这是由于甲醇为质子接受体溶剂,乙腈为质子给予体溶剂,二者在分离选择性上有一定的差异^[28,29]^。由图2可以看出,以甲醇为有机相时,分析时间长达20 min,而以乙腈为有机相时,在10 min内5种分析物即可完全分离。这是由于与甲醇相比,乙腈的极性更小,会增强对分析物的洗脱强度,从而使保留时间缩短^[29]^。根据Empower 3色谱工作站的计算结果,虽然以甲醇或乙腈为有机相时,5种分析物均能完全分开(分离度大于2),但分离度最小的ADB-BUTINACA和4F-MDMB-BUTICA在乙腈为有机相时的分离效果更好,且以乙腈为有机相时,色谱峰明显更窄。因此,最终选择乙腈作为有机相。

**图2:**
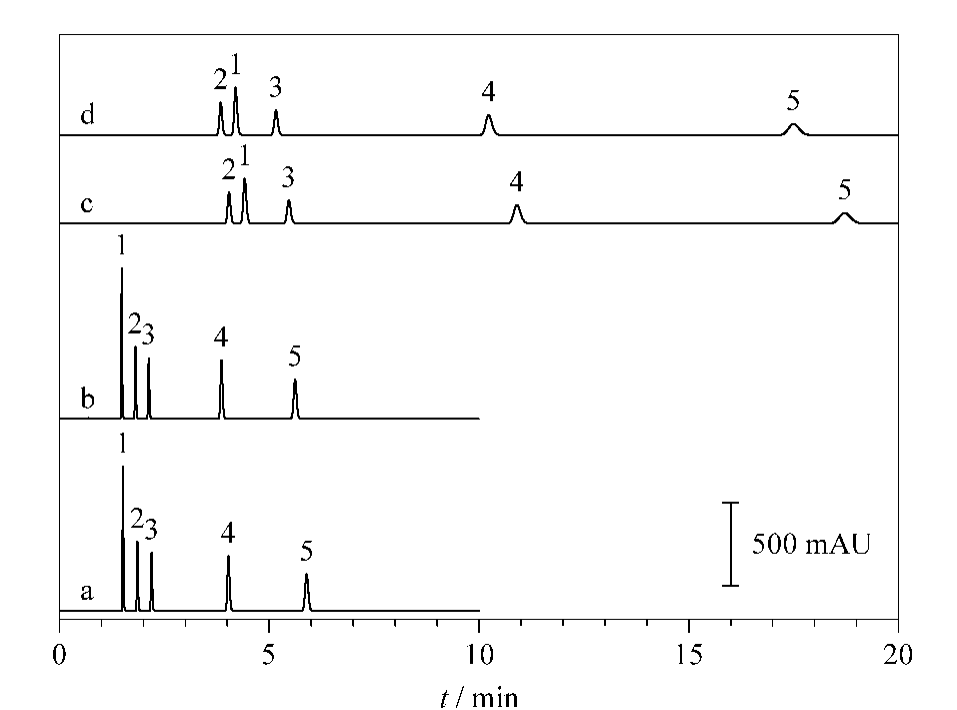
不同流动相下5种合成大麻素的色谱图

分别以超纯水和磷酸三乙胺缓冲溶液为水相进行分析。使用超纯水时,5个色谱峰的保留时间比使用磷酸三乙胺时长1.8%~4.9%,而分离度、峰面积、峰高、峰宽等并无明显差异。考虑到使用缓冲盐易导致液相色谱流路堵塞,因此最终选择超纯水作为水相。

#### 2.1.3 洗脱程序的优化

由图2可以看出,采用乙腈-超纯水(65∶35, v/v)进行等度洗脱时,前3种分析物在色谱柱上的保留时间较短。为了使5种分析物的保留时间较好地分布在梯度时间内,对有机相比例进行调整。采用不同体积比的乙腈-超纯水(65∶35、 60∶40、 50∶50, v/v)进行等度洗脱,从图3可知,随着有机相比例的降低,分析物的保留时间延长,但色谱峰也随运行时间的增加而展宽,不利于检测和准确定量。因此,采用梯度洗脱方式对5种分析物进行同时分离。

**图3:**
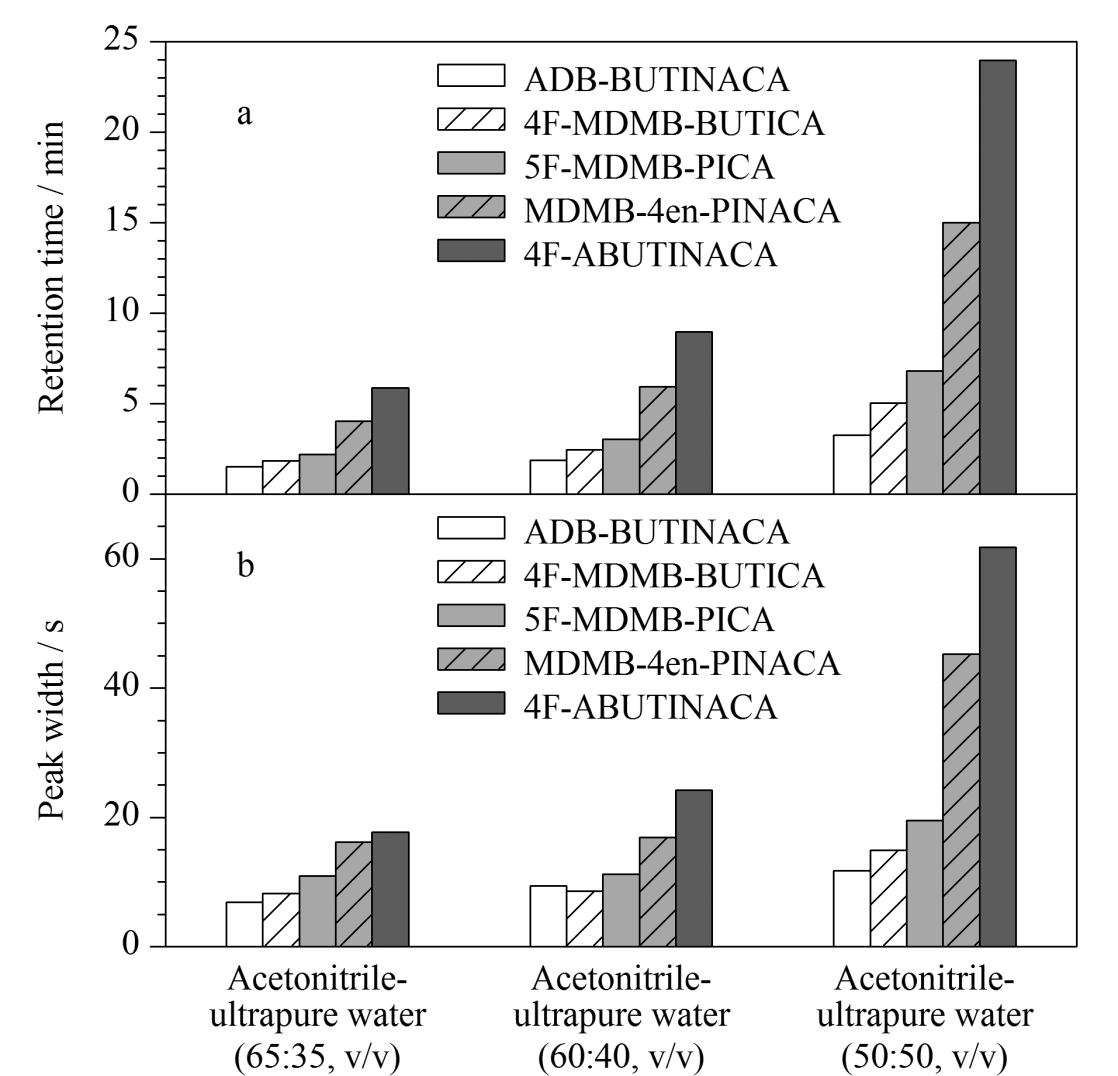
等度洗脱程序下有机相比例对5种合成大麻素(a)保留时间和(b)峰宽的影响

根据等度洗脱结果,在10 min内进行50%~100%乙腈超纯水溶液的线性梯度洗脱(流速为0.3 mL/min)。在梯度洗脱开始时流动相的洗脱能力较弱,与固定相亲和力较低的ADB-BUTINACA、4F-MDMB-BUTICA和5F-MDMB-PICA在色谱柱上有一定的保留;随着有机相比例逐渐增加,与固定相亲和力较高的MDMB-4en-PINACA和4F-ABUTINACA被更快地洗脱下来,5种分析物可实现完全分离。为了使色谱柱在运行过程中能够更好地平衡,对上述线性梯度洗脱程序进行微调,调整后的梯度洗脱程序见表1。

#### 2.1.4 柱温的影响

采用表1中的梯度洗脱程序对5种分析物进行分离检测,分别考察柱温为25、30、35、40 ℃时对色谱分离的影响。在不同柱温条件下,相邻分析物的分离度均大于9,可充分满足定量分析需求。此外,随着柱温的升高,5种分析物的保留时间减少1%~6%。当柱温为35 ℃时,5种分析物的色谱峰更窄,峰宽可比另外3种柱温条件小1%~8%。因此,综合考虑上述因素,最终将柱温设为35 ℃。

在优化的色谱条件下,5种合成大麻素的色谱图见图4,其中ADB-BUTINACA、MDMB-4en-PINACA和4F-ABUTINACA的检测波长为302 nm, 4F-MDMB-BUTICA和5F-MDMB-PICA的检测波长为290 nm。

**图4:**
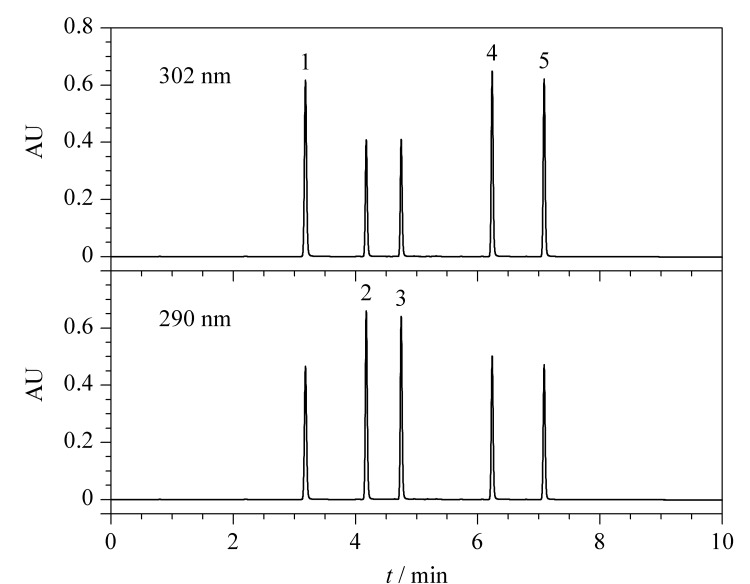
5种合成大麻素在检测波长分别为302 nm和290 nm下的液相色谱图

### 2.2 方法学评价

#### 2.2.1 线性关系、检出限与定量限

按照1.2节配制不同质量浓度的系列混合标准溶液进行测定,以各组分的质量浓度(*x*, mg/L)为横坐标,峰面积(*y*)为纵坐标绘制曲线,曲线采用线性拟合,权重1/*x*。结果显示,5种合成大麻素在1~100 mg/L范围内线性关系良好,相关系数均可以达到0.9999。检出限的计算和表达方法较多,根据《化学分析方法验证确认和内部质量控制实施指南色谱分析》(GB/T 35655-2017)^[30]^,本研究将使用逐步稀释法得到的结果作为检出限。将添加了5种合成大麻素标准物质的空白烟油样品提取溶液进行逐步稀释,得到系列低质量浓度的样品,进行测定。将信噪比(*S/N*)为3时的信号值所对应的质量浓度视为检出限,将*S/N*=10时的信号值所对应的质量浓度视为定量限^[25,26,31]^。结果如表2所示,5种合成大麻素的检出限为0.2 mg/L,定量限为0.6 mg/L,方法的灵敏度可以满足日常检测需求。

**表2:** 5种合成大麻素的保留时间、检测波长、线性方程、相关系数(*r*^2^)、检出限及定量限

Compound	Retention time/min	Detection wavelength/nm	Regression equation	*r*^2^	LOD/ (mg/L)	LOQ/ (mg/L)
ADB-BUTINACA	3.157	302	*y*=7.49×10^3^*x*+6.82×10^2^	0.9999	0.2	0.6
4F-MDMB-BUTICA	4.145	290	*y*=7.22×10^3^*x*+6.40×10^2^	0.9999	0.2	0.6
5F-MDMB-PICA	4.711	290	*y*=6.66×10^3^*x*+6.22×10^2^	0.9999	0.2	0.6
MDMB-4en-PINACA	6.172	302	*y*=6.78×10^3^*x*+6.98×10^2^	0.9999	0.2	0.6
4F-ABUTINACA	7.003	302	*y*=6.61×10^3^*x*+8.06×10^2^	0.9999	0.2	0.6

*y*: peak area; *x*: mass concentration, mg/L.

#### 2.2.2 准确度

本研究以回收试验确定准确度,并参照GB/T35655-2017^[30]^对准确度进行评价。向不含分析物的空白电子烟油样品中添加5种合成大麻素的混合标准溶液,在低(2 mg/L)、中(10 mg/L)、高(50 mg/L)3个水平下进行加标回收试验,每个加标水平平行测定6次,计算回收率。结果表明,各分析物的平均加标回收率为95.5%~101.9%,相对标准偏差(RSD, *n*=6)为0.2%~1.5%。参考中国药典四部(2020年版)^[31]^中样品中待测定成分含量和回收率的限度关系,本研究各加标水平下的回收率均符合要求。5种合成大麻素的平均加标回收率和RSD见表3。同时参照GB/T 35655-2017^[30]^计算准确度,并与通常的准确度范围(-20%~10%)^[30]^进行比较。由表3可以看出,各加标水平下分析数据的准确度均符合要求。

**表3:** 5种合成大麻素的平均加标回收率、相对标准偏差和准确度(*n*=6)

Compound	Spiked level/ (mg/L)	Recovery/ %	RSD/ %	Accuracy/ %
ADB-BUTINACA	2	95.6	1.5	-4.4
	10	101.6	0.3	1.6
	50	97.6	0.2	-2.4
4F-MDMB-BUTICA	2	96.1	0.6	-4.0
	10	101.3	0.4	1.3
	50	97.1	0.2	-2.9
5F-MDMB-PICA	2	96.9	1.0	-3.1
	10	101.6	0.3	1.6
	50	96.8	0.2	-3.2
MDMB-4en-PINACA	2	96.7	0.5	-3.3
	10	101.9	0.7	1.9
	50	97.3	0.2	-2.7
4F-ABUTINACA	2	95.5	0.5	-4.5
	10	101.4	0.4	1.4
	50	96.8	0.3	-3.2

#### 2.2.3 精密度

制备质量浓度分别为1、10、100 mg/L的5种合成大麻素混合标准溶液,每个质量浓度平行测定6次,计算得到日内精密度(intra-day RSD);连续测定6天,计算得到日间精密度(inter-day RSD),相关数据见表4。由表4可知,5种合成大麻素的日内精密度均小于1.5%,日间精密度均小于2.2%。由结果可知,该方法的精密度良好^[31]^。

**表4:** 5种合成大麻素在3个加标水平下的日内、日间精密度(*n*=6)

Compound	1 mg/L						10 mg/L						100 mg/L			
	Intra-day RSD/%		Inter-day RSD/%				Intra-day RSD/%		Inter-day RSD/%				Intra-day RSD/%		Inter-day RSD/%	
ADB-BUTINACA		0.73		1.20				0.33		0.52				0.25		0.55
4F-MDMB-BUTICA		0.82		1.54				0.42		0.80				0.34		0.58
5F-MDMB-PICA		0.50		1.25				0.22		0.71				0.33		0.51
MDMB-4en-PINACA		0.49		1.15				0.28		0.50				0.34		0.41
4F-ABUTINACA		1.43		2.13				0.64		0.67				0.42		0.47

### 2.3 实际样品检测

利用该方法对案件中缴获的电子烟油样品进行分析。按1.3节方法对样品进行预处理后,再根据1.4节的色谱条件对样品进行分析,两个样品的色谱图见图5。与标准物质色谱图进行比对,采用外标法定量,确定1号样品中检出合成大麻素5F-MDMB-PICA(检测波长290 nm),含量为0.45%(质量分数); 确定2号样品中检出合成大麻素ADB-BUTINACA、MDMB-4en-PINACA、4F-ABUTINACA(检测波长均为302 nm),含量分别为0.059%、0.071%、0.15%(质量分数)。由检测结果可知,在实际样品分析中,各分析物的分离效果好,方法准确、可靠。

**图5:**
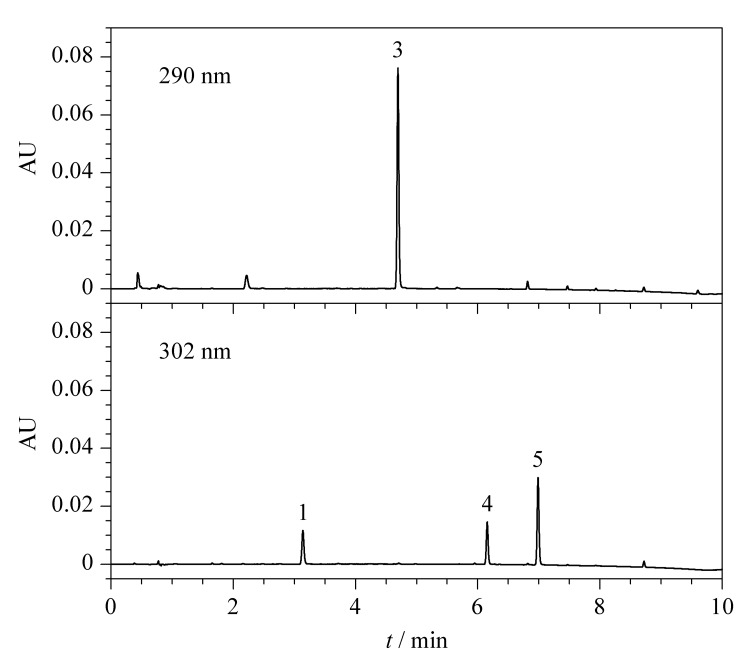
两个电子烟油样品的色谱图

## 3 结论

本文建立了超高效液相色谱同时测定电子烟油中5种吲哚/吲唑酰胺类合成大麻素的分析方法,该法可在10 min内将5种合成大麻素完全分离并进行定量分析。经过实际样品验证,本方法具有准确、快速、灵敏、分离效果好等优点,适用于电子烟油中5种吲哚/吲唑酰胺类合成大麻素的定量分析,能够满足相关鉴定工作的要求,为相关案件中合成大麻素的鉴定提供参考。
